# Engineering the Structure–Performance Relationship of WO_3_ Thin Films Through PVP–Mn Synergy for Electrochromic Applications

**DOI:** 10.3390/polym18202542

**Published:** 2026-10-16

**Authors:** Pritam J. Morankar, Sureshkumar Kandhasamy, Thamaraiselvi Kanagaraj, Chan-Wook Jeon

**Affiliations:** 1 School of Chemical Engineering, Yeungnam University, 280 Daehak-ro, Gyeongsan 38541, Republic of Korea; pritam.nanoworld@gmail.com; 2 Department of Chemistry, KIT-Kalaignar Karunanidhi Institute of Technology (Autonomous), Coimbatore 641402, Tamil Nadu, India; ksuresh022@gmail.com; 3 Advanced Functional Materials Laboratory, Department of Orthodontics, Saveetha Dental College and Hospitals, Saveetha Institute of Medical and Technical Sciences, Chennai 600077, Tamil Nadu, India; tsthamarai89@gmail.com

**Keywords:** PVP–Mn synergy, Mn doping, PVP-assisted electrodeposition, electrochromism, Li^+^ ion transport, optical modulation, smart windows

## Abstract

The electrochromic (EC) behavior of tungsten oxide (WO_3_) is strongly influenced by its nanoscale architecture and local chemical environment, which determine the efficiency of ion–electron transport during switching. Here, a systematic polyvinylpyrrolidone (PVP)–Manganese (Mn) synergistic engineering approach was developed using bare WO_3_ (WO), PVP-assisted WO_3_ (P-WO), Mn-doped WO_3_ (Mn-WO), and PVP-assisted Mn-doped WO_3_ (P-Mn-WO) to clarify the individual and combined effects of PVP and Mn on EC performance. All films retained the amorphous WO_3_ structure, while morphological evolution revealed a transition from compact nanoparticle assemblies to an interconnected porous architecture in P-Mn-WO. The optimized P-Mn-WO film exhibited enhanced Li^+^ transport, with diffusion coefficients of 0.45 × 10^−10^ and 4.08 × 10^−10^ cm^2^/s for oxidation and reduction, respectively. This favorable transport environment enabled 75.8% optical modulation at 635 nm, a coloration efficiency of 142.63 cm^2^/C, and 92.59% charge reversibility, with only 5.24% degradation during repeated switching. At the device level, P-Mn-WO delivered 82.30% bleached transmittance, 12.02% colored transmittance, and 70.28% optical modulation, while showing only 4.5% degradation over 2000 s of continuous operation. These results demonstrate that coupling PVP-assisted growth with Mn doping provides effective control over WO_3_ morphology, Li^+^ transport, charge utilization, and optical switching, establishing a promising route toward a durable, high-contrast WO_3_-based EC smart-window.

## 1. Introduction

Globally, buildings account for a substantial proportion of total energy consumption, making the efficient regulation of solar heat gain and daylight through building envelopes a key strategy for improving energy efficiency [1]. However, conventional glazing provides limited adaptability to continuously changing environmental conditions, creating a growing demand for window technologies capable of dynamically controlling light transmission. EC smart windows have emerged as one of the most promising solutions because their optical transmittance can be reversibly modulated by a small electrical stimulus, enabling on-demand regulation of daylight and solar heat gain [2]. For practical applications, however, EC materials must simultaneously exhibit high optical modulation, rapid coloration and bleaching, high coloration efficiency (CE), and excellent cycling stability [3]. Achieving these performance characteristics requires efficient ion–electron transport within the EC layer, making precise control over the material’s structural and chemical characteristics essential [4].

WO_3_ is a representative cathodic EC material owing to its reversible ion insertion/extraction, high transparency in the bleached state, suitable redox chemistry, and compatibility with thin-film configurations [5]. Nevertheless, the EC response of WO_3_ remains strongly dependent on its structural and physicochemical characteristics [6]. Zhao et al. reported amorphous WO_3_ films exhibiting 64.4% optical modulation, bleaching/coloring times of 1.2/3.1 s, and a CE of 42.5 cm^2^/C, which was attributed to favorable ion-transport pathways [7]. Xie et al. further demonstrated that the crystallinity and oxygen-vacancy concentration of WO_3_ strongly influence its EC characteristics, while excessive crystallization can induce ion trapping and incomplete bleaching [8]. These findings clearly indicate that the EC characteristics of WO_3_ are governed not only by its chemical composition but also by the local structural environment through which ions and electrons are transported [9]. Consequently, rational engineering of this environment has become an important strategy for improving WO_3_-based EC materials [10].

Among the various material-engineering approaches, polymer-assisted modification has emerged as a simple yet effective strategy for tailoring the growth and functional characteristics of metal-oxide thin films [11]. Functional polymers can interact with precursor species and developing oxide surfaces, influencing nucleation, growth, interfacial characteristics, and the accessibility of electrochemically active regions [12]. Among them, PVP is particularly attractive because its polar carbonyl-containing pyrrolidone groups can interact with metal-containing species during WO_3_ formation [13]. Such interactions can modify the development of the oxide network and consequently influence the pathways available for electrolyte interaction and ion transport. Thus, incorporating PVP provides a means of improving the functional characteristics of WO_3_ while preserving its fundamental EC chemistry [14]. However, modification of film formation alone may not fully address the electronic and redox characteristics that govern charge compensation during ion insertion/extraction [15].

To further optimize the intrinsic electronic and redox characteristics of WO_3_, elemental incorporation has emerged as an effective complementary strategy. Introducing foreign metal ions into the WO_3_ lattice can regulate the local coordination environment, defect concentration, and charge-transfer behavior, thereby facilitating ion–electron transport during EC switching [16]. Zhou et al. reported Ni-doped WO_3_ films exhibiting >85% optical modulation, CE of 60.5 cm^2^/C, and excellent cycling stability over approximately 5500 cycles [7]. Mak et al. achieved 54.9% optical modulation using 3.9% Nb-doped WO_3_, whereas lower Nb concentrations provided superior long-term cycling stability [17]. Sun et al. further demonstrated dual-band electrochromism in Ti-doped WO_3_·2H_2_O, achieving 83.8% and 72.5% optical modulation at 633 and 1050 nm, respectively [18]. These studies demonstrate that elemental engineering provides an additional degree of control over the EC behavior of WO_3_ by tailoring its intrinsic chemical and electronic characteristics [19].

Considering that polymer assistance and elemental incorporation regulate different aspects of WO_3_, integrating these two approaches offers a logical pathway toward further performance enhancement [20]. While PVP primarily governs film formation and the local electrochemical environment, elemental dopants modify the intrinsic electronic structure and redox behavior of the WO_3_ framework. Among various transition-metal dopants, Mn is particularly attractive because of its multiple oxidation states, which can effectively influence the electronic structure and charge-transfer characteristics of metal oxides. Therefore, coupling PVP-assisted growth with Mn incorporation is expected to provide complementary regulation of both the structural and electronic characteristics of WO_3_, thereby promoting more efficient ion–electron transport during EC switching. However, despite this potential, the individual contributions of PVP and Mn, as well as their synergistic influence on the EC performance of WO_3_, have not yet been systematically investigated.

To address this gap, we designed a controlled four-film system comprising bare WO, P-WO_,_ Mn-WO, and P-Mn-WO. This design enables the effects of PVP and Mn to be distinguished individually while providing a direct basis for evaluating their combined contribution. The films were fabricated by electrodeposition and systematically examined using structural, morphological, compositional, and chemical characterization, followed by electrochemical and EC evaluation. The resulting correlations between material modification and optical modulation, CE, switching kinetics, and cycling stability provide insight into the role of combined PVP and Mn engineering in regulating the EC behavior of WO_3_**,** offering a rational pathway toward high-performance EC thin films for smart-window applications.

## 2. Experimental Section

### 2.1. Materials

Sodium tungstate dihydrate (Na_2_WO_4_·2H_2_O, Sigma-Aldrich U.S., St. Louis, MO, USA), hydrogen peroxide (H_2_O_2_, 30 wt%, Sigma-Aldrich U.S., St. Louis, MO, USA), nitric acid (HNO_3_, Sigma-Aldrich U.S., St. Louis, MO, USA), polyvinylpyrrolidone (PVP, average molecular weight ≈ 40,000, Sigma-Aldrich U.S., St. Louis, MO, USA), and manganese (II) sulfate monohydrate (MnSO_4_·H_2_O, Sigma-Aldrich U.S., St. Louis, MO, USA) were purchased from commercial suppliers and used as received without further purification. Fluorine-doped tin oxide (FTO, MTI, Seoul, Republic of Korea)-coated glass substrates were used as the conducting substrates for thin-film deposition. Acetone, ethanol, and deionized (DI) water were used for substrate cleaning and solution preparation.

### 2.2. Preparation of WO_3_-Based Thin Films

Prior to electrodeposition, the FTO substrates were sequentially ultrasonically cleaned in acetone, ethanol, and deionized water for 15 min each, followed by drying under a nitrogen stream. The precursor solution was prepared by dissolving 0.05 mol of Na_2_WO_4_·2H_2_O in 100 mL of DI under continuous magnetic stirring. Subsequently, 10 mL of H_2_O_2_ was added dropwise to form a transparent peroxotungstate solution, followed by the addition of a few drops of HNO_3_ to stabilize the precursor. The solution was maintained at 50 °C under constant stirring to promote the formation of a stable peroxotungstate complex, facilitating controlled nucleation and uniform growth of WO_3_ thin films.

To systematically investigate the individual and combined effects of polymer assistance and Mn incorporation, four deposition electrolytes were prepared. The pristine precursor solution was used for the deposition of the WO film. For the P-WO sample, 0.30 g of PVP was dissolved in the precursor solution under continuous stirring until a homogeneous solution was obtained. The Mn-WO electrolyte was prepared by adding 0.30 g of MnSO_4_·H_2_O to the pristine precursor solution. For the P-Mn-WO sample, 0.15 g of PVP and 0.15 g of MnSO_4_·H_2_O were simultaneously introduced into the precursor solution while maintaining identical preparation conditions. All precursor solutions were magnetically stirred until completely homogeneous prior to deposition. Electrodeposition was performed in a conventional three-electrode electrochemical cell using the cleaned FTO substrate as the working electrode, a platinum mesh as the counter electrode, and an Ag/AgCl electrode as the reference electrode. WO_3_-based thin films were deposited by cyclic voltammetry within a potential window of +1.0 to −1.0 V for 25 deposition cycles. After deposition, the films were thoroughly rinsed with deionized water to remove residual electrolyte and dried under ambient conditions (80 °C for 12 h) before subsequent characterization.

### 2.3. Electrochromic Device Fabrication

Based on its superior EC performance, P-Mn-WO was selected as the active electrode for device fabrication. The EC cell was constructed in a Glass/FTO/P-Mn-WO/1 M LiClO_4_–PC/FTO/Glass sandwich configuration, with bare FTO serving as the counter electrode. A homogeneous 1 M LiClO_4_/PC electrolyte was introduced between the electrodes and confined using a transparent double-sided adhesive spacer (Scotch Brand Tape, 3M, St. Paul, MN, USA) to ensure a uniform electrode separation. The two electrodes were aligned face-to-face, and the device perimeter was subsequently sealed to minimize electrolyte leakage and solvent evaporation. The assembled device was then used to evaluate the practical optical modulation and long-term EC stability of the optimized P-Mn-WO electrode.

### 2.4. Materials Characterization and Electrochemical Measurements

The structural evolution of the prepared WO_3_-based thin films was investigated by X-ray diffraction (XRD, PANalytical, Almelo, The Netherlands) to identify their crystallographic characteristics. Changes in surface architecture were examined using field-emission scanning electron microscopy (FESEM, S-4800, Hitachi, Tokyo Japan), while the elemental composition and spatial distribution of the constituent elements were verified by energy-dispersive X-ray spectroscopy (EDS). The surface chemical composition together with the oxidation states of W, Mn, and O were analyzed using X-ray photoelectron spectroscopy (XPS, K-Alpha, Thermo Fisher Scientific, Cheshire, UK). The electrochemical and EC properties of the deposited films were evaluated using an IVIUM CompactStat.e electrochemical workstation (IVIUM Technologies, Eindhoven, The Netherlands). Measurements were carried out in a standard three-electrode configuration, where the WO, P-WO, Mn-WO, and P-Mn-WO films deposited on FTO glass served as the working electrodes, while a platinum wire and an Ag/AgCl electrode were employed as the counter and reference electrodes, respectively. 1 M LiClO_4_ dissolved in PC was used as the electrolyte throughout the electrochemical experiments. The EC behavior was systematically assessed by cyclic voltammetry (CV), and chronocoulometry (CC). Simultaneous optical and electrochemical responses during the coloration and bleaching processes were monitored using a UV–Vis spectrophotometer (S-3100, SCINCO, Seoul, Republic of Korea) coupled with the electrochemical workstation. The optical modulation (ΔT), coloration efficiency (CE), switching kinetics (coloration and bleaching times), charge density, and cycling durability were subsequently determined to establish the influence of PVP-assisted growth and Mn incorporation on the EC performance of the WO_3_ thin films.

## 3. Results and Discussions

### 3.1. XRD Analysis

**Figure 1:**
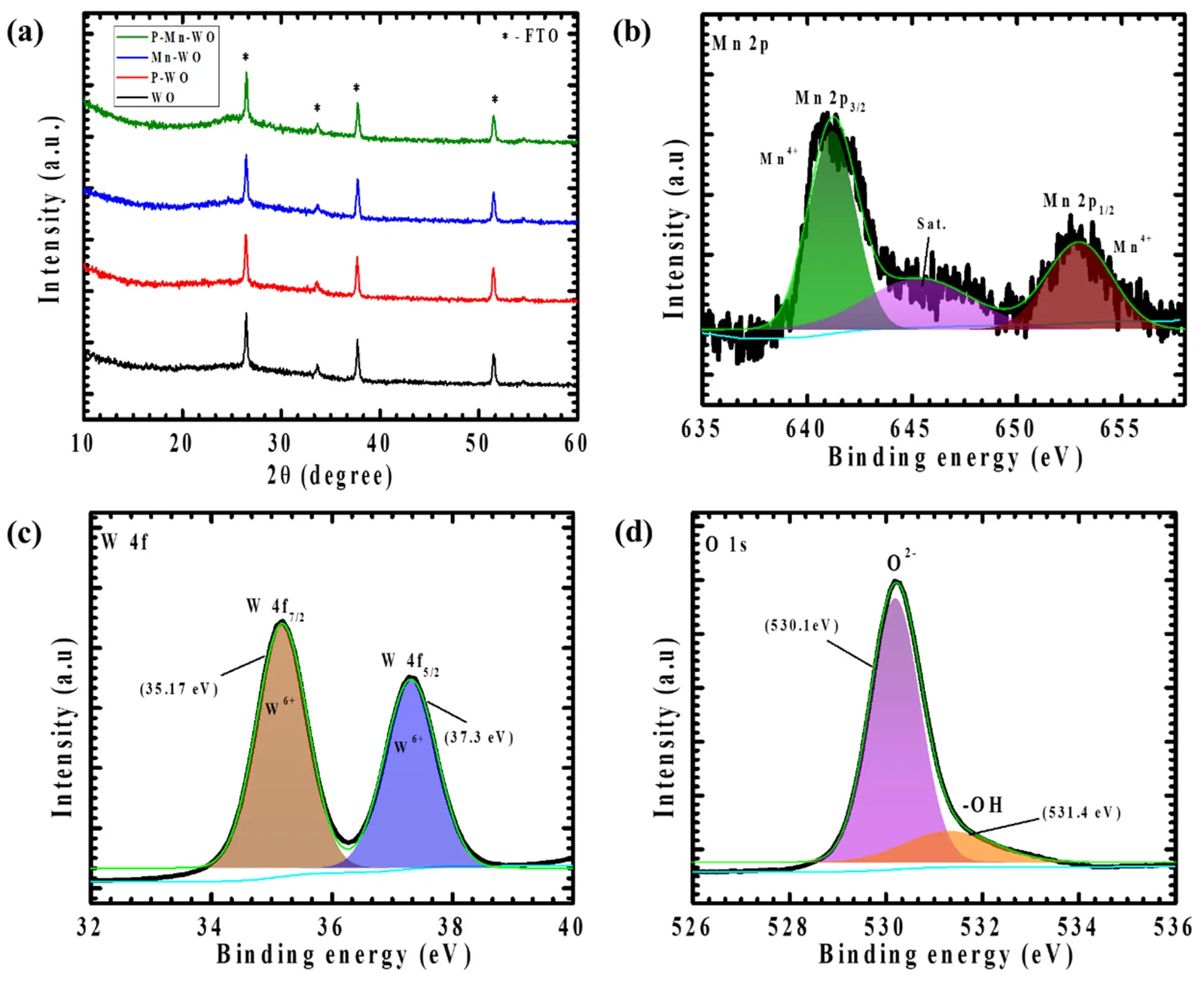
(**a**) XRD patterns of WO, P-WO, Mn-WO, and P-Mn-WO thin films, high-resolution XPS spectra of the optimized P-Mn-WO thin film: (**b**) Mn, (**c**) W 4f, and (**d**) O 1s.

The XRD patterns of WO, P-WO, Mn-WO, and P-Mn-WO thin films were presented in Figure 1a. All samples show nearly identical diffraction profiles, indicating that PVP assistance and Mn incorporation do not significantly change the structural nature of the WO_3_ films. The prominent peaks at approximately 26.6°, 33.5°, 37.9°, and 51.8° originate from the FTO substrate. In contrast, no distinct diffraction peaks corresponding to crystalline WO_3_ are observed. A broad diffuse halo appears in the lower 2θ region, confirming the amorphous nature of the deposited WO_3_ films [21]. This broad feature indicates short-range atomic ordering without long-range crystallographic periodicity. The amorphous character is retained for all four samples, including the PVP-assisted and Mn-incorporated films. This confirms that PVP and Mn do not induce crystallization during the electrodeposition process. No additional peaks associated with Mn-based crystalline phases are detected. Therefore, Mn incorporation does not produce any detectable secondary crystalline phase within the XRD detection limit. Similarly, PVP does not introduce any new phase into the WO_3_ matrix. The almost overlapping diffraction profiles demonstrate that the phase composition remains essentially unchanged after modification. Thus, PVP and Mn primarily influence the local structure and deposition characteristics rather than the overall phase structure. The preserved amorphous framework is advantageous for EC applications. Its disordered network can provide accessible pathways for Li^+^ insertion and extraction during electrochemical switching. Therefore, the XRD results confirm that the modified films maintain a phase-pure amorphous WO_3_ structure. The EC improvements can consequently be attributed mainly to microstructural and local chemical modifications rather than crystallographic transformation [22].

### 3.2. XPS Elucidation

The chemical composition and electronic states of the optimized P-Mn-WO thin film were investigated by XPS, and the corresponding high-resolution spectra are presented in Figure 1b–d. The W 4f spectrum in Figure 1b exhibits two distinct components corresponding to W 4f_7/2_ and W 4f_5/2_, confirming the predominant W^6+^ oxidation state in the WO_3_ framework. The well-resolved doublet and characteristic spin–orbit separation indicate that tungsten remains mainly in the oxidized state after PVP-assisted Mn incorporation. The Mn spectrum in Figure 1c confirms the successful presence of Mn in the optimized film. The deconvoluted Mn-related components suggest that Mn exists in different local chemical environments within the WO_3_ matrix, which can modify the electronic distribution around the W–O bonds [23]. Such incorporation can introduce local lattice distortion and influence the redox activity of the oxide network. The O 1s spectrum shown in Figure 1d consists of a dominant contribution from lattice oxygen associated with W–O bonding, together with higher-binding-energy components related to oxygen-deficient regions and surface-adsorbed oxygen species. The presence of these additional oxygen environments indicates a non-uniform local oxygen coordination in the P-Mn-WO film. Oxygen-deficient sites can act as defect centers and modify the electronic structure of WO_3_, while also providing favorable sites for interaction with electrolyte ions. The combined presence of Mn and oxygen-related defects is therefore expected to facilitate charge redistribution and improve the accessibility of active sites during electrochemical operation. The XPS results confirm the successful incorporation of Mn into the W**^6+^** based WO_3_ framework and reveal multiple oxygen environments associated with the modified surface. These chemical features provide a reasonable basis for the enhanced Li^+^ insertion/extraction, charge-transfer kinetics, and EC switching observed for the optimized P-Mn-WO film [24].

### 3.3. Morphological and Elemental Compositional Characteristics

**Figure 2:**
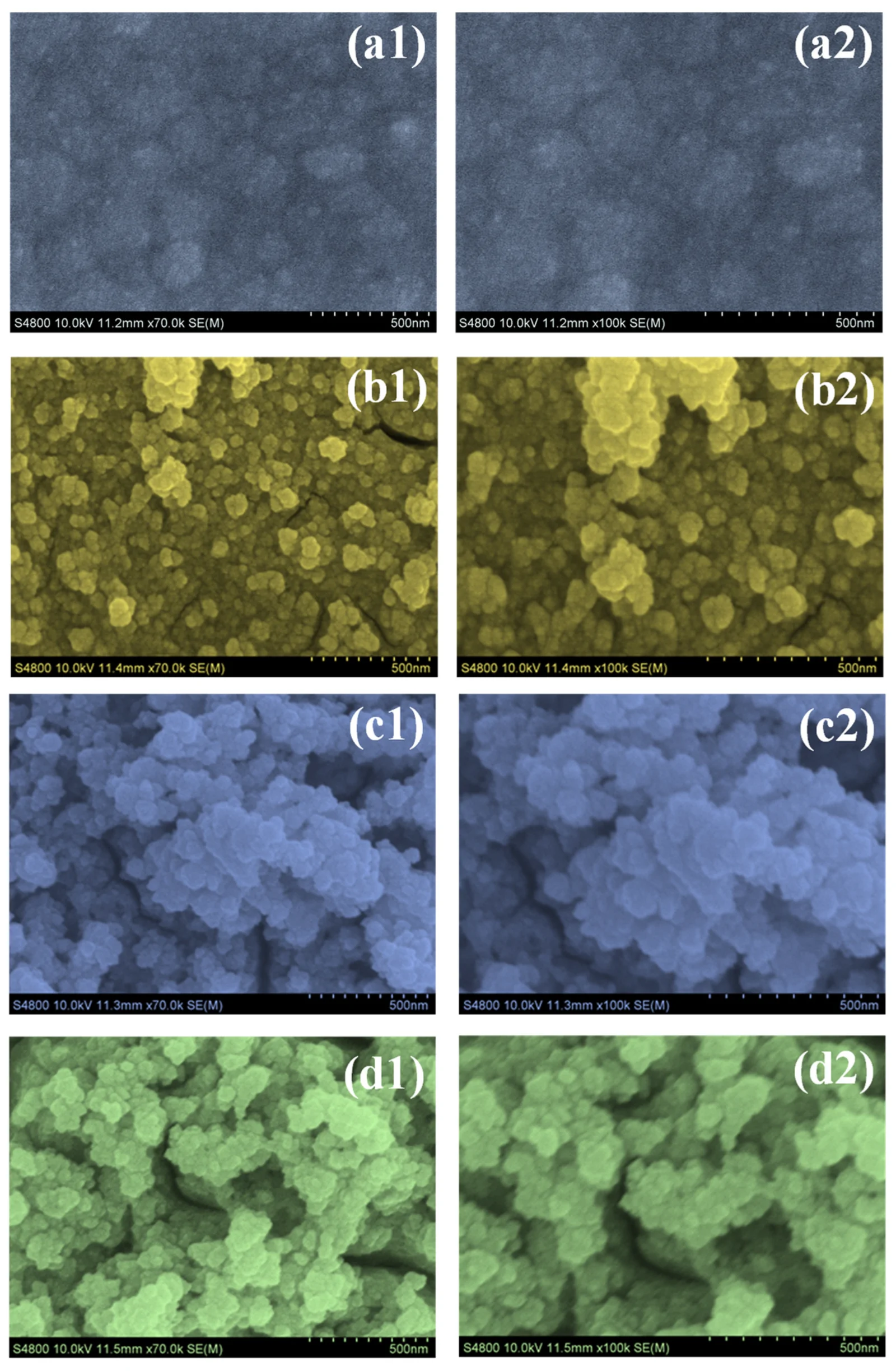
FESEM images of (**a1**,**a2**) WO, (**b1**,**b2**) P-WO, (**c1**,**c2**) Mn-WO, and (**d1**,**d2**) P-Mn-WO thin films.

The surface morphologies of the WO, P-WO, Mn-WO, and P-Mn-WO thin films were investigated by FESEM, as presented in Figure 2. A distinct and progressive evolution in the surface architecture is observed with the sequential incorporation of PVP and Mn, confirming their influence on the nucleation and growth behavior of electrodeposited WO_3_. The WO thin film in Figure 2(a1,a2) exhibits a compact and relatively smooth surface composed of densely packed ultrafine nanoparticles [25]. The nanoparticles are strongly agglomerated with poorly defined boundaries, resulting in a dense film with limited interparticle voids, which suggests rapid nucleation and relatively uncontrolled particle growth. Following PVP incorporation, the P-WO thin film shown in Figure 2(b1,b2) exhibits uniformly distributed spherical nanograins with reduced agglomeration and improved surface homogeneity. This morphological evolution can be attributed to the interaction of the carbonyl groups of PVP with tungsten precursor species, which regulates nucleation and suppresses excessive particle coalescence during deposition [26]. Consequently, a finer and more uniformly distributed nanoparticulate structure is obtained. Further modification with Mn produces a distinct structural transformation in the Mn-WO film, as observed in Figure 2(c1,c2). In contrast to the relatively dispersed nanograins of P-WO, Mn-WO consists of interconnected nanoparticle aggregates forming a continuous three-dimensional framework with abundant intergranular spaces. This indicates that Mn modifies the nucleation kinetics and promotes the assembly of WO_3_ nanoparticles into an interconnected porous architecture. The most pronounced morphological evolution is observed for P-Mn-WO in Figure 2(d1,d2), where a highly uniform hierarchical network composed of interconnected nanoparticle clusters and well-developed porous channels is formed. The improved particle distribution and interparticle connectivity indicate a synergistic effect of PVP and Mn, where PVP regulates particle growth while Mn promotes nanoparticle assembly. Overall, the progressive morphological evolution from WO → P-WO → Mn-WO → P-Mn-WO, as observed in Figure 2, demonstrates the complementary roles of PVP and Mn in tailoring the WO_3_ microstructure. The resulting interconnected and porous architecture can enhance electrolyte accessibility and provide favorable pathways for Li^+^ ion transport, thereby facilitating reversible EC reactions. Consequently, the optimized morphology of P-Mn-WO provides a favorable structural basis for its improved EC performance [27].

**Figure 3:**
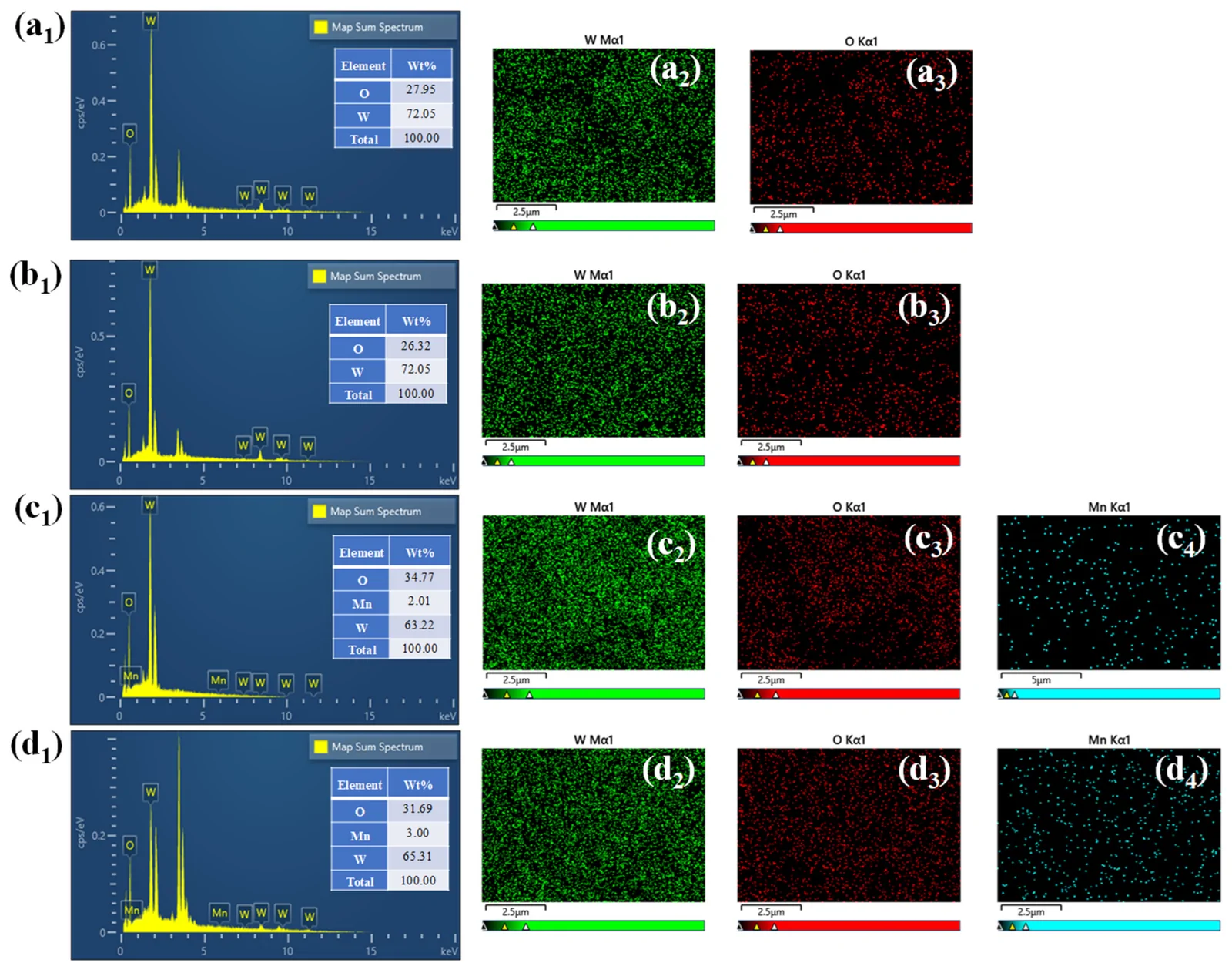
(**a_1_**–**d_1_**) EDS spectra and (**a_2_**–**d_4_**) elemental mapping of WO, P-WO, Mn-WO, and P-Mn-WO thin films.

The elemental composition and spatial distribution of WO, P-WO, Mn-WO, and P-Mn-WO thin films were investigated using EDS and elemental mapping, as shown in Figure 3. The WO film exhibits W and O contents of 72.05 and 27.95 wt%, respectively (Figure 3(a_1_)), confirming the formation of the WO_3_ matrix, while the corresponding O and W maps (Figure 3(a_2_,a_3_)) demonstrate their uniform distribution throughout the film. For P-WO, the EDS spectrum shows W (72.05 wt%) and O (26.32 wt%), with homogeneous elemental distributions observed in the corresponding maps (Figure 3(b_1_–b_3_)). The Mn-WO film confirms the presence of W, O, and Mn with contents of 63.22, 34.77, and 2.01 wt%**,** respectively (Figure 3(c_1_)), while the W, O, and Mn maps (Figure 3(c_2_–c_4_)) indicate uniform Mn incorporation within the WO_3_ matrix. Similarly, P-Mn-WO contains 65.31 wt% W, 31.69 wt% O, and 3.00 wt% Mn (Figure 3(d_1_)), with the corresponding elemental maps (Figure 3(d_2_–d_4_)) showing a homogeneous distribution without noticeable elemental segregation. The EDS and mapping results confirm the compositional uniformity of the deposited films and successful Mn incorporation, supporting the synergistic role of PVP and Mn in modifying the WO_3_ structure for enhanced electrochromic performance.

To further assess the morphological characteristics of the deposited films, cross-sectional FESEM analysis was performed to determine their thicknesses (Figure S1a–d). The WO, P-WO, Mn-WO, and P-Mn-WO films exhibited thicknesses of approximately 550, 580, 605, and 620 nm, respectively. The relatively moderate variation in thickness confirms that all films were formed within a comparable submicrometer thickness range. Although the P-Mn-WO film is slightly thicker, its enhanced EC performance is not considered to originate solely from the thickness difference. Instead, the improved optical modulation and ion transport are associated with the combined influence of PVP-assisted film growth, Mn incorporation, and the interconnected porous morphology developed in the P-Mn-WO film.

## 4. Electrochromic Analysis

**Figure 4:**
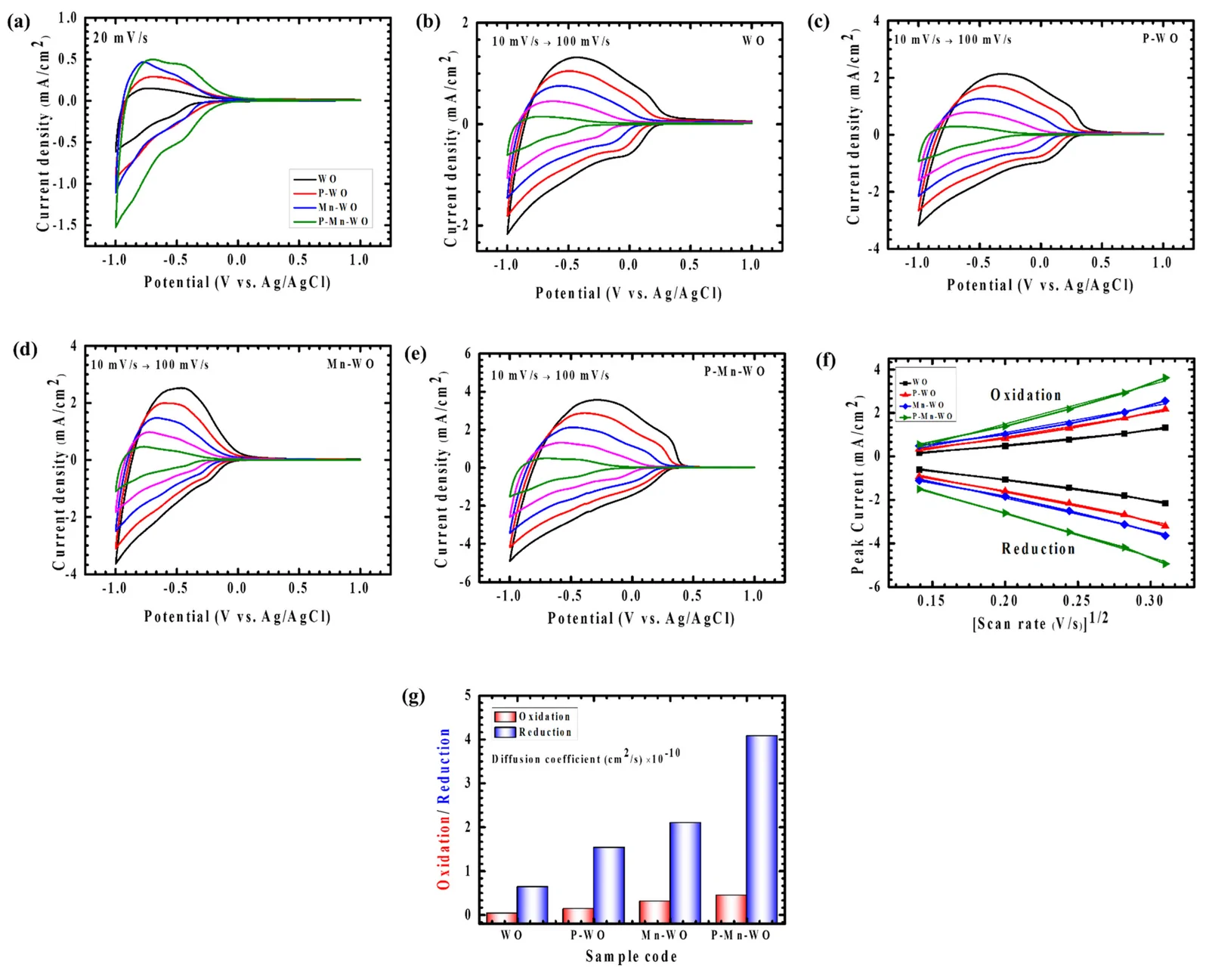
Electrochemical characteristics of WO, P-WO, Mn-WO, and P-Mn-WO thin films: (**a**) CV profiles recorded at 20 mV/s; (**b**–**e**) CV profiles recorded at scan rates of 20–100 mV/s; (**f**) linear relationship between peak current and the square root of scan rate; and (**g**) comparison of Li^+^ diffusion coefficients during oxidation and reduction processes at 20 mV/s.

The electrochemical characteristics of the WO, P-WO, Mn-WO, and P-Mn-WO thin films were investigated by cyclic voltammetry to understand the ion–electron processes responsible for their EC switching behavior. The CV profiles recorded at 20 mV/s, presented in Figure 4a, show distinct oxidation and reduction responses for all films, confirming the reversible electrochemical activity of the WO_3_-based electrodes. These redox processes are associated with the coupled insertion/extraction of Li^+^ ions and electrons, which drives the reversible transition between the colored and bleached states of WO_3_. The bare WO film shows a comparatively weak electrochemical response, whereas PVP assistance and Mn incorporation progressively increase the CV response [28]. Among the investigated electrodes, P-Mn-WO exhibits the largest enclosed CV area and strongest redox currents, indicating a higher electrochemically active contribution during the EC switching process. The enhanced response suggests that the combined PVP and Mn modification improves the accessibility of the WO_3_ active regions and promotes more efficient ion–electron exchange during coloration and bleaching. To examine the EC reaction kinetics under different operating rates, CV measurements were performed at scan rates ranging from 20 to 100 mV/s, as shown in Figure 4b–e. The anodic and cathodic currents increase progressively with scan rate for all films, while the characteristic redox features remain identifiable throughout the investigated range. The evolution of the CV profiles reflects the ability of the WO_3_ layers to accommodate Li^+^ insertion and extraction under increasingly rapid potential changes. The pristine WO electrode shows a comparatively limited response at higher scan rates, whereas P-WO and Mn-WO exhibit improved redox activity [29]. In particular, P-Mn-WO maintains the strongest electrochemical response across the entire scan-rate range, indicating more favorable kinetics for the ion-coupled redox reactions associated with EC switching. The improved rate response can be attributed to the interconnected nanoparticle network developed through PVP-assisted growth and Mn incorporation, which facilitates electrolyte penetration and provides accessible pathways for Li^+^ migration throughout the electrochromically active layer [30].

To further clarify the origin of the rate-dependent response, the variation in peak current (*i*_p_) with the square root of scan rate (ν^1/2^) was analyzed in Figure 4f. The approximately linear relationship between *i*_p_ and ν^1/2^ indicates that Li^+^ diffusion within the WO_3_ film is an important factor governing the electrochemical response. The Li^+^ diffusion coefficient was calculated using the Randles–Sevcik equation (Equation (1)):

(1) $$D^{\frac{1}{2}}\;=\frac{i_{p}}{2.69\times 10^{5}\times n^{3/2}\times A\times C\times \vartheta^{1/2}}$$

where *i*_p_ is the peak current, *n* is the number of electrons involved in the redox process, *A* is the electrochemically active area of the working electrode, *D* is the Li^+^ diffusion coefficient, *C* is the Li^+^ concentration in the electrolyte, and ν is the scan rate. For consistency, the diffusion coefficients of all four electrodes were determined from the CV data recorded at 20 mV/s, and the calculated values are summarized in Table 1. The corresponding comparison is also presented in Figure 4g. The optimized P-Mn-WO electrode exhibits the highest Li^+^ diffusion coefficients, reaching 0.45 × 10^−10^ cm^2^/s for oxidation and 4.08 × 10^−10^ cm^2^/s for reduction, confirming its more favorable ion-transport characteristics during the EC process. The higher reduction-side diffusivity indicates particularly favorable Li^+^ insertion into the WO_3_ framework during coloration, while the oxidation value represents Li^+^ extraction associated with the bleaching process [31]. The enhanced Li^+^ mobility can be correlated with the interconnected and porous morphology of P-Mn-WO, which provides improved electrolyte access and continuous pathways for ion migration. Efficient ion transport through this structure facilitates the reversible ion–electron coupling required for EC switching. Therefore, the diffusion analysis provides further evidence that the PVP-assisted Mn modification effectively regulates the WO_3_ structure and promotes Li^+^ transport, contributing to the superior EC activity of the P-Mn-WO thin film [32].

**Figure 5:**
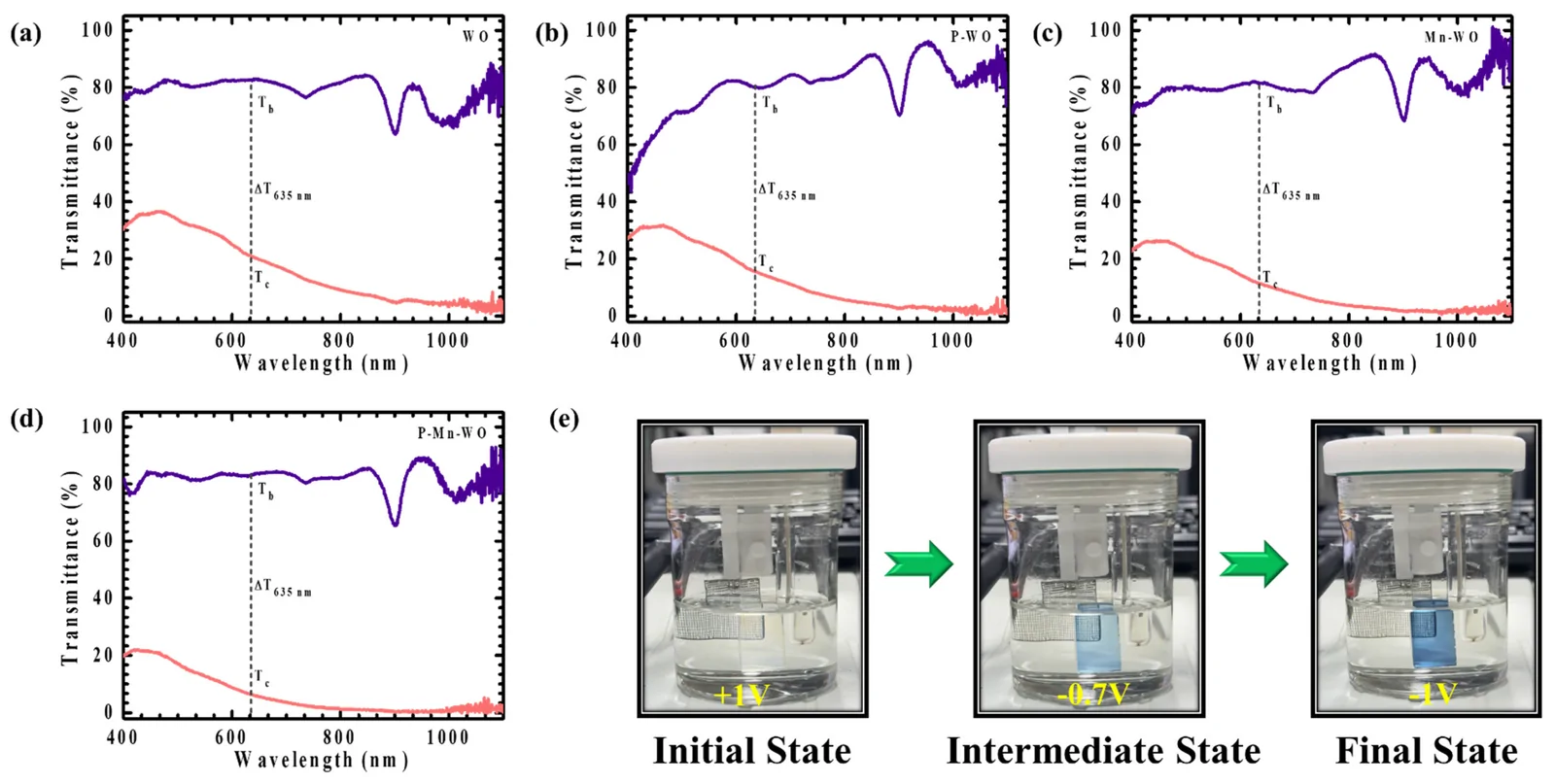
In situ optical transmittance characteristics of WO, P-WO, Mn-WO, and P-Mn-WO thin films: transmittance spectra of (**a**) WO, (**b**) P-WO, (**c**) Mn-WO, and (**d**) P-Mn-WO in the bleached and colored states; (**e**) digital photographs of P-Mn-WO thin film at different applied potentials.

The EC properties of the WO, P-WO, Mn-WO, and P-Mn-WO thin films were evaluated by in situ UV–Vis spectroscopy using a 1 M LiClO_4_/PC electrolyte, as shown in Figure 5. The transmittance variation during electrochemical Li^+^ insertion/extraction was used to assess the coloration and bleaching characteristics of the films [33]. Here, the bleached-state transmittance (T_b_) corresponds to the transparent state, whereas the colored-state transmittance (T_c_) represents the transmittance after coloration. The optical modulation was calculated as ΔT = T_b_ − T_c_. The corresponding transmittance spectra are presented in Figure 5a–d, where all films exhibit a clear and reversible change in optical transmittance between the bleached and colored states. The optical parameters measured at 635 nm are summarized in Table 2. The WO film exhibits a T_b_ of 82.6% and a T_c_ of 20.9%, corresponding to an optical modulation of 61.7%. With PVP assistance, the P-WO film shows a lower Tc of 15.6% while maintaining a relatively high T_b_ of 80.2%, resulting in an improved ΔT of 64.6%. Mn incorporation produces a further enhancement, with the Mn-WO film exhibiting T_b_ = 81.4%, T_c_ = 11.5%, and ΔT = 69.9%. Notably, the combined PVP-assisted Mn-doped film (P-Mn-WO) provides the highest EC contrast, with T_b_ = 82.1%, T_c_ = 6.3%, and ΔT = 75.8%. The progressive increase in ΔT from 61.7% for WO to 75.8% for P-Mn-WO indicates that the simultaneous incorporation of PVP and Mn is more effective than either modification alone. In addition to visible-light modulation, near-infrared (NIR) optical modulation is important for evaluating EC materials for smart-window applications because it reflects their ability to regulate NIR transmission associated with solar heat. The NIR optical modulation was calculated as the difference between the transmittance of the bleached and colored states at 1050 nm. The calculated NIR optical modulation values were 76.75% (Tb-80.3%; Tc-3.55%), 80.22% (Tb-82.8%; Tc-2.58%), 81.01% (Tb-82.91%; Tc-1.9%), and 82.70% (Tb-83.74%; Tc-1.04%) for WO, PVP-WO, Mn-WO, and PVP-Mn-WO, respectively. Notably, PVP-Mn-WO exhibited the highest NIR optical modulation, owing to its combination of high bleached-state transmittance (83.74%) and low colored-state transmittance (1.04%). These results demonstrate its enhanced ability to modulate NIR transmission within the measured spectral range, highlighting its potential for EC smart-window applications. In particular, the low Tc of P-Mn-WO demonstrates its ability to achieve deep coloration without compromising the transparency of the bleached state [34]. The potential-dependent optical response of P-Mn-WO is shown in Figure 5e. The film remains highly transparent at +1 V, whereas the transmittance gradually decreases as the applied potential becomes more negative. A visible blue coloration appears at −0.7 V and becomes substantially darker at −1 V, demonstrating the controllable optical switching of the film. The potential-dependent optical response of P-Mn-WO can be understood in terms of the reversible electrochemical insertion and extraction of Li^+^ ions accompanied by electron transfer within the WO_3_ framework. During coloration, the applied negative potential promotes Li^+^ insertion and electron injection, resulting in the partial reduction of W^6+^ to W^5+^ and the formation of lithium tungsten bronze (Li_x_WO_3_), as described by Li_x_WO_3_ ⇌ WO_3_ + xLi^+^ + xe^−^, with the forward direction corresponding to coloration under this convention. The coexistence of W^6+^ and W^5+^ sites facilitates localized electronic transitions, including polaron-related absorption and intervalence charge transfer, which contribute to the broad optical absorption and the development of the blue-colored state. Upon reversing the applied potential, Li^+^ ions and electrons are extracted, the tungsten species become reoxidized, and the film returns toward its transparent bleached state. The extent of coloration therefore depends on the reversible charge insertion process and the associated changes in the electronic structure of WO_3_. The improved optical modulation of P-Mn-WO may be associated with the combined influence of PVP-assisted film formation and Mn incorporation. PVP may promote more uniform particle distribution and influence the film microstructure, potentially facilitating electrolyte access to electrochemically active regions. Mn incorporation may modify the local coordination environment and electronic structure of the WO_3_ matrix, thereby influencing its electrochemical response. When combined, these modifications may favour effective Li^+^ insertion and extraction and enhance the utilization of the WO_3_ film during coloration. These proposed contributions are consistent with the enhanced optical modulation observed for P-Mn-WO [35].

**Figure 6:**
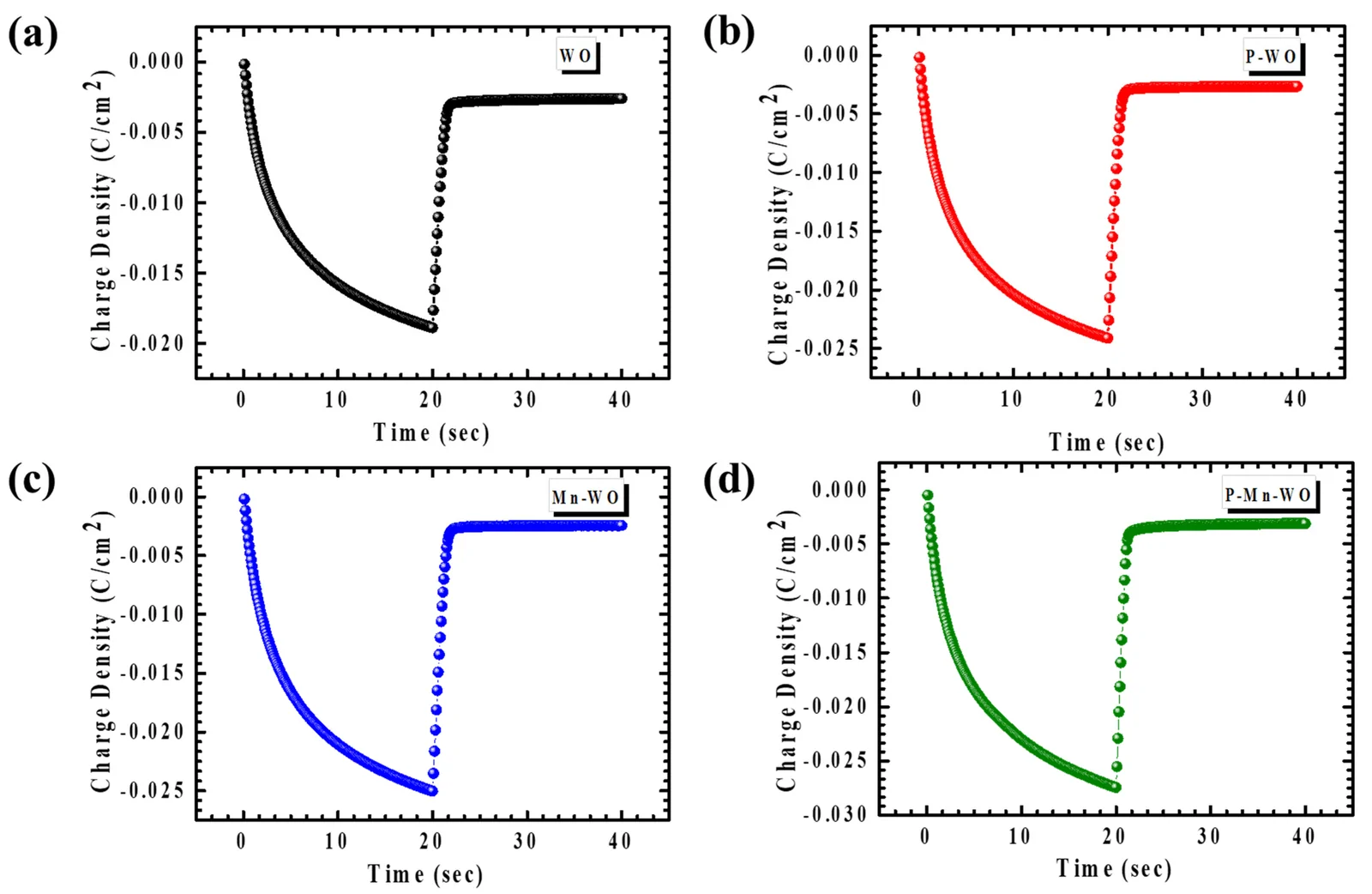
Chronocoulometric responses of (**a**) WO, (**b**) P-WO, (**c**) Mn-WO, and (**d**) P-Mn-WO thin films.

The charge-storage behavior of the deposited films was evaluated by chronocoulometry (CC) to determine how effectively the charge introduced during coloration could be recovered during bleaching [36]. The corresponding responses for WO, P-WO, Mn-WO, and P-Mn-WO are shown in Figure 6a–d. During the cathodic coloration process, Li^+^ ions enter the WO_3_ film with simultaneous electron injection, whereas the reverse polarization drives Li^+^ out of the film and restores the bleached state. Thus, comparison of the charge accumulated during these two steps provides a useful measure of the reversibility of the electrochemical reaction. The reversibility was calculated from the intercalation charge (Q_i_) and deintercalation charge (Q_di_) using Equation (2):

(2) $$\mathrm{Reversibility}\;=\;\frac{Qdi}{Qi}\times 100$$

A clear improvement in charge recovery is observed across the four films. The WO film shows Q_i_ and Q_di_ values of 0.018 and 0.015 C/cm^2^, respectively, corresponding to a reversibility of 83.33%. With PVP assistance, the P-WO film accommodates a higher charge of 0.022 C/cm^2^, while 0.019 C/cm^2^ is recovered during deintercalation, increasing the reversibility to 86.36%. Mn-WO exhibits a further increase in charge exchange, with Q_i_ = 0.025 C/cm^2^ and Q_di_ = 0.022 C/cm^2^, resulting in 88.10% reversibility. The P-Mn-WO film shows the most pronounced response, reaching an intercalation charge of 0.027 C/cm^2^ and recovering 0.025 C/cm^2^ during deintercalation. Accordingly, its reversibility reaches 92.59%, the highest value among the investigated films. The difference between Q_i_ and Q_di_ becomes progressively smaller with the modification of WO_3_, indicating more effective recovery of the charge introduced during coloration. This trend is particularly evident for P-Mn-WO, where most of the intercalated charge is released during the subsequent deintercalation step. The result suggests that PVP assistance and Mn incorporation improve the accessibility of the electrochemically active WO_3_ network and facilitate Li^+^ transport during repeated insertion/extraction. Rather than simply increasing the amount of charge involved in the reaction, the combined modification also improves the fraction of charge that can be reversibly exchanged. The CC results are in good agreement with the optical switching behavior. P-Mn-WO, which exhibits the highest charge reversibility of 92.59%, also provides the largest optical modulation of 75.8%. The simultaneous improvement in charge exchange and optical contrast indicates that the PVP/Mn modification creates a more favorable environment for the reversible ion–electron reactions responsible for WO_3_ electrochromism. Therefore, the enhanced charge reversibility of P-Mn-WO is an important factor behind its superior EC response and demonstrates the effectiveness of the combined PVP-assisted Mn modification [37].

The coloration efficiency (CE) provides a more meaningful assessment of the EC response by relating the optical density change to the amount of charge involved during coloration [38]. In other words, it indicates how effectively the electrochemically supplied charge is utilized to produce the optical transition. The CE values of WO, P-WO, Mn-WO, and P-Mn-WO were calculated at 635 nm from the optical density change between the bleached and colored states according to Equation (3):

(3) CE = ΔODQiA = lnTbTcQiA

where T_b_ and T_c_ denote the transmittance in the bleached and colored states, respectively, and Q_i_ is the charge density involved in the coloration process. The calculated CE values are summarized in Table 2 and show a progressive improvement with the modification of the WO_3_ films. The pristine WO film exhibits a CE of 76.11 cm^2^/C, which increases to 106.36 cm^2^/C for P-WO following PVP assistance. With Mn incorporation, the CE further increases to 117.42 cm^2^/C for Mn-WO, while the combined PVP and Mn modification produces the highest value of 142.63 cm^2^/C for P-Mn-WO. Accordingly, the CE follows the order WO < P-WO < Mn-WO < P-Mn-WO, confirming the beneficial effect of the successive modifications on charge utilization during EC switching. The increase in CE indicates that a larger optical density change is achieved from the charge involved in the coloration process. The improvement is particularly pronounced for P-Mn-WO, suggesting that the combined modification provides a more favorable electrochemical environment for Li^+^ transport and charge compensation within the WO_3_ layer. The modified nanoparticle architecture can enhance electrolyte contact and facilitate ion movement through the electroactive film, thereby promoting a more efficient conversion of electrical charge into optical contrast. In addition, the higher CE indicates that the enhanced EC response is not solely associated with increased charge insertion, but also with more effective utilization of the inserted charge. This behavior agrees with the high charge reversibility observed for P-Mn-WO, further supporting its efficient ion–electron exchange during switching. The highest CE of 142.63 cm^2^/C, combined with an optical modulation of 75.8%, demonstrates that P-Mn-WO provides an excellent balance between charge utilization and optical contrast. Therefore, the synergistic contribution of PVP assistance and Mn incorporation is responsible for the improved EC efficiency of the optimized P-Mn-WO film [39].

**Figure 7:**
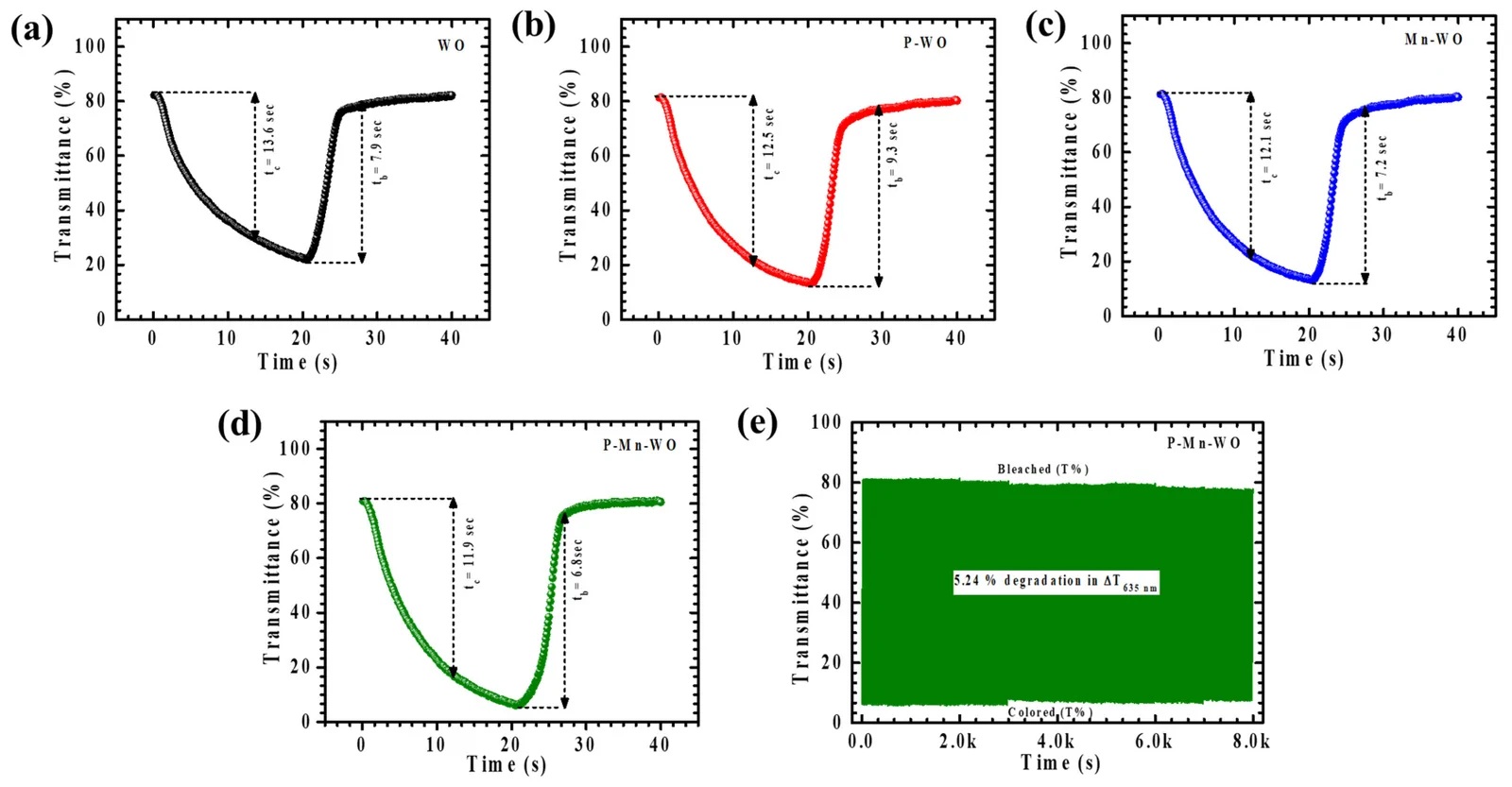
Real-time EC switching characteristics of (**a**) WO, (**b**) P-WO, (**c**) Mn-WO, and (**d**) P-Mn-WO thin films under alternating potentials of −1.0 and +1.0 V with a pulse duration of 20 s; (**e**) cycling stability of the P-Mn-WO thin film during repeated coloration/bleaching cycles.

The dynamic optical response of the WO, P-WO, Mn-WO, and P-Mn-WO films was examined at 635 nm to determine their switching behavior, as shown in Figure 7a–d. The measurements were performed in 1 M LiClO_4_/PC electrolyte by alternately applying −1.0 and +1.0 V for 20 s. The coloration and bleaching times were calculated at 95% of the total transmittance change, and the resulting values are listed in Table 2. Each film exhibits a distinct decrease in transmittance under negative polarization followed by recovery when the positive potential is applied, confirming the reversible nature of the EC process [40]. The transmittance profiles become progressively more favorable after PVP assistance and Mn incorporation. Compared with WO, the modified films show a more pronounced and responsive optical transition, with P-Mn-WO exhibiting the most effective switching behavior. The faster response of the modified films can be associated with the changes in the WO_3_ film structure during deposition. PVP-assisted growth and Mn incorporation produce a more favorable nanoparticle arrangement, allowing the electrolyte to interact more effectively with the active WO_3_ surface. This facilitates Li^+^ movement during both insertion and extraction and consequently improves the rate of the coloration/bleaching process. The switching response of P-Mn-WO is further supported by its high charge reversibility of 92.59%, indicating efficient recovery of the electrochemically exchanged charge.

For practical EC applications, maintaining the optical response during repeated switching is as important as achieving a fast initial response. Therefore, the cycling durability of the optimized P-Mn-WO film was separately evaluated by continuously monitoring its transmittance at 635 nm, as shown in Figure 7e. The P-Mn-WO electrode maintains a stable and repeatable transmittance response during successive coloration and bleaching cycles, with only 5.24% degradation in ΔT after the stability test. The limited decrease in optical modulation indicates that the film can withstand repeated Li^+^ insertion/extraction while retaining most of its initial EC activity. The combined results demonstrate that the P-Mn-WO film offers a favorable balance between switching response and durability. Its 75.8% optical modulation, 142.63 cm^2^/C coloration efficiency, and 92.59% charge reversibility, together with only 5.24% ΔT degradation, indicate efficient and sustained electrochemical switching. These characteristics confirm that the simultaneous use of PVP and Mn is an effective approach for improving the practical EC performance of WO_3_ thin films [41].

To further assess the EC performance of the P-Mn-WO film, a comparison with representative WO_3_-based EC systems reported in the literature is presented in Table 3 [42,43,44,45,46,47,48,49]. The reported materials include doped WO_3_ binary WO_3_-based systems, and WO_3_ composites prepared using different deposition approaches. The P-Mn-WO film exhibits an optical modulation of 75.8% at 635 nm and a coloration efficiency of 142.63 cm^2^/C, which represents a favorable combination of optical modulation and coloration efficiency compared with several reported WO_3_-based systems. In addition, the P-Mn-WO film maintains stable EC switching for 8000 s with only 5.24% degradation. Although some reported systems exhibit higher optical modulation or longer cycling stability, the P-Mn-WO film provides a balanced EC response arising from the combined effects of PVP-assisted morphological regulation and Mn incorporation. The direct comparison of EC performance is influenced by differences in film thickness, measurement wavelength, electrolyte, applied potential, electrode configuration, and stability-testing conditions. Therefore, the comparison primarily serves to demonstrate the competitive and balanced performance of the present P-Mn-WO system within the broader WO_3_-based EC literature.

## 5. Electrochromic Device Performance

**Figure 8:**
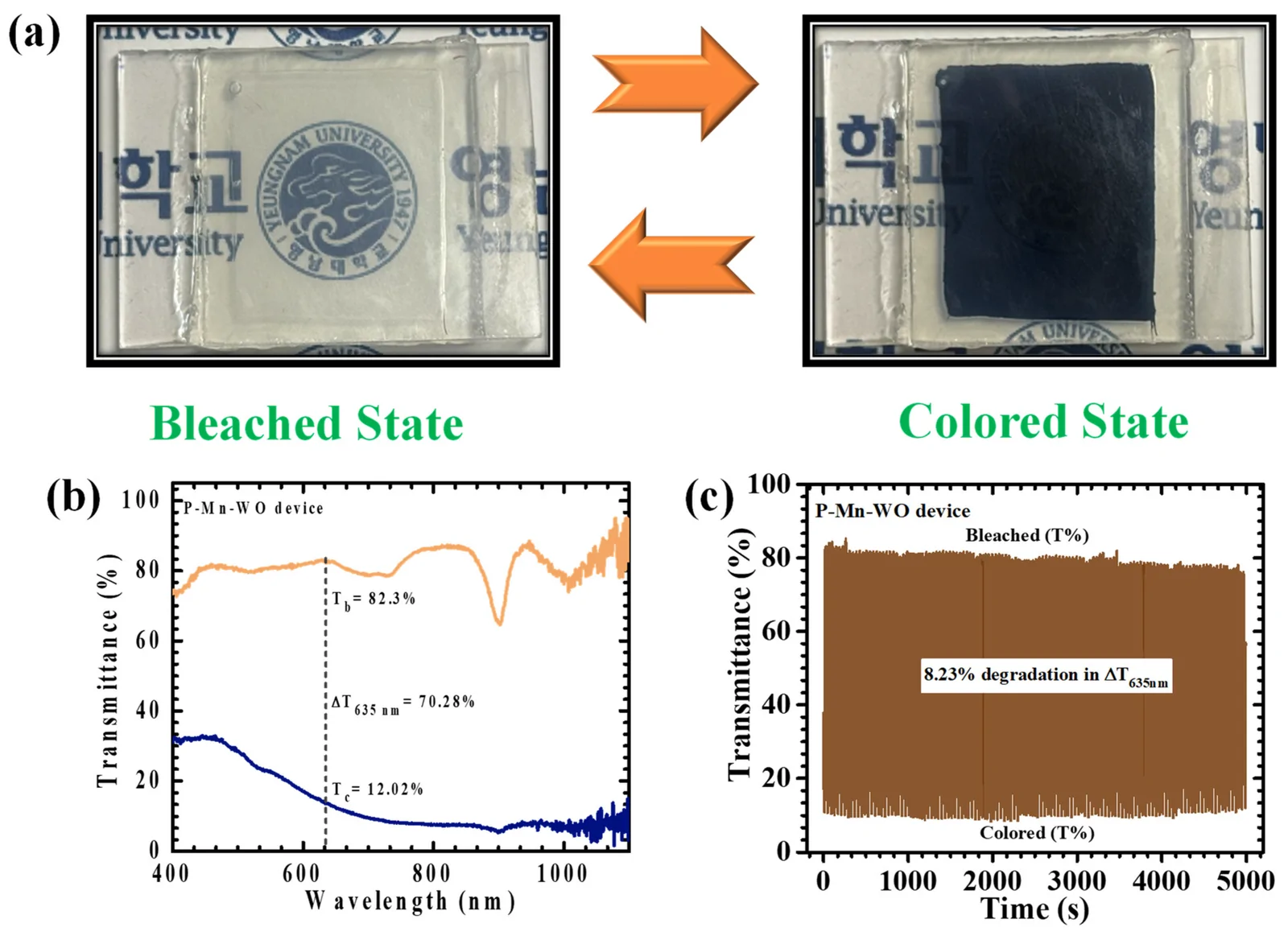
EC device performance of the optimized P-Mn-WO electrode: (**a**) digital photographs of the device in the bleached and colored states; (**b**) optical transmittance spectra of the device in the bleached and colored states; and (**c**) cycling stability of the device at 635 nm during continuous operation for 5000 s.

The practical EC behavior of the optimized P-Mn-WO electrode was further examined by assembling a sandwich-type device consisting of P-Mn-WO/FTO/1 M LiClO_4_/PC/FTO glass. The device response in the bleached and colored states is visually presented in Figure 8a. Upon applying the coloring potential, the initially transparent device develops a distinct colored appearance, while reversing the potential restores the transparent state. The pronounced change in appearance occurs reproducibly, indicating that the optical switching characteristics of the P-Mn-WO film are effectively maintained after device fabrication. The optical response of the device was quantified from the transmittance spectra shown in Figure 8b. In the bleached state, the device exhibits a high transmittance of 82.30% (T_b_), whereas the transmittance decreases to 12.02% (T_c_) after coloration. The resulting ΔT of 70.28% reflects a substantial change in the amount of transmitted light between the two optical states. Such a large separation between T_b_ and T_c_ is favorable for EC windows because the device can simultaneously provide high light transmission in the bleached state and pronounced light blocking in the colored state. The observed modulation results from the reversible Li^+^ ion exchange between the electrolyte and the P-Mn-WO layer, which induces the corresponding optical change in the WO framework. The stability of the device was monitored continuously for 5000 s, as shown in Figure 8c. Repeated coloration and bleaching produced well-defined transmittance changes throughout the measurement, while the optical response decreased by only 8.23% from its initial value. The limited loss of modulation indicates that the device maintains its electrochemical activity during prolonged switching. In particular, the preservation of the optical response suggests that the P-Mn-WO layer and the electrolyte interface remain sufficiently stable under repeated Li^+^ insertion and extraction, preventing rapid deterioration of the device response.

To complement the optical assessment, post-cycling FESEM characterization was performed on P-Mn-WO, and the corresponding images are presented in Figure S2a,b. These images enable an examination of the surface morphology after repeated switching and provide complementary structural information for evaluating the durability of the active film. The FESEM images reveal minor morphological changes, including localized cracking and partial deformation of some nanospheres, following repeated coloration–bleaching cycles. Nevertheless, the overall nanoparticle-based surface architecture remains largely preserved, without evidence of extensive structural collapse. These observations suggest that the P-Mn-WO film maintains reasonable morphological integrity during the extended stability test, despite localized changes induced by repeated electrochemical switching. The observed structural preservation is consistent with the relatively stable EC response recorded over long-term duration.

## 6. Conclusions

In this work, WO, P-WO, Mn-WO, and P-Mn-WO thin films were developed to clarify the synergistic effect of PVP and Mn on EC performance. All films retained an amorphous structure, while PVP assistance and Mn incorporation progressively modified the film morphology, with P-Mn-WO exhibiting an interconnected nanoparticle network favorable for Li^+^ transport. The optimized P-Mn-WO showed Li^+^ diffusion coefficients of 0.45 × 10^−10^ and 4.08 × 10^−10^ cm^2^/s for oxidation and reduction, respectively. At 635 nm, it achieved 75.8% ΔT, 142.63 cm^2^/C CE, and 92.59% charge reversibility, with only 5.24% degradation during repeated switching. The corresponding EC device exhibited T_b_ and T_c_ values of 82.30% and 12.02%, respectively, yielding a ΔT of 70.28%, while retaining stable optical switching with only 4.5% degradation over 2000 s. These results demonstrate that the combined PVP–Mn modification effectively regulates the structural and electrochemical characteristics of WO_3_, resulting in enhanced optical contrast and operational stability. The progressive improvement from WO to P-Mn-WO confirms the advantage of simultaneously controlling film architecture and local chemical characteristics. This strategy provides a practical pathway for developing durable, high-performance WO_3_-based EC devices for smart-window applications.

## Figures and Tables

**Table 1:** Calculated diffusion coefficients of WO, P-WO, Mn-WO, and P-Mn-WO thin films.

Sample Code	Diffusion Coefficient (cm^2^/s) × 10^−10^	
	Oxidation	Reduction
WO	0.04	0.64
P-WO	0.14	1.54
Mn-WO	0.30	2.10
P-Mn-WO	0.45	4.08

**Table 2:** Evaluation of EC measurements of WO, P-WO, Mn-WO, and P-Mn-WO thin films.

Sample Code	Charge Intercalation (Q_i_) (C/cm^2^)	Charge Deintercalation (Q_di_) (C/cm^2^)	Reversibility (%)	Coloration Time (s) (t_c_)	Bleaching Time (s) (t_b_)	T_b_ %	T_c_ %	Optical Modulation (ΔT_635nm_%)	Coloration Efficiency (cm^2^/C)
WO	0.018	0.015	83.33	13.6	7.9	82.6	20.9	61.7	76.11
P-WO	0.022	0.019	86.36	12.5	9.3	80.2	15.6	64.6	106.36
Mn-WO	0.025	0.022	88.1	12.1	7.2	81.4	11.5	69.9	117.42
P -Mn-WO	0.027	0.025	92.59	11.9	6.8	82.1	6.3	75.8	142.63

**Table 3:** Comparison of the electrochromic properties of WO_3_-based materials reported in the literature.

Sr. No.	Material	Method	Morphology	ΔT%	Coloration Efficiency (cm^2^/C)	Stability	Ref.
1.	WO_3_/MoO_3_	Spray pyrolysis	Nanoparticles	31% (430 nm)	40.9	400 s	[42]
2.	Sm-WO_3_/ Polydopamine	Hydrothermal	Nanoclusters	68.5% (633 nm)	72.7	5000 cycles	[43]
3.	Gd-WO_3_	Co-sputtering	Nano-stalagmite	52.7% (633 nm)	74.7	2000 cycles	[44]
4.	Nd–Mo co-doped SnO_2_/α-WO_3_	Dip coating/ Electrodeposition	Hexagonal framework	90% (600 nm)	200	1000 cycles	[45]
5.	WO_3_-V_2_O_5_	electrochemical co-deposition	Agglomerated particles	60% (700 nm)	61.5	1000 s	[46]
6.	Gd-WO_3_/Ag/WO_3_	Sputtering	Nanoparticles	49.6% (633 nm)	52.1	2000 cycles	[47]
7.	P-WO_3_	Electrodeposition	Nanograins	80.8% (550 nm)	53	1000 cycles	[48]
8.	P-CQD/a-WO_3_	hydrothermal/ spin coating	Nanoparticles	62.5% (633 nm)	76.1	4000 cycles	[49]
9.	P-Mn-WO	Electrodeposition	interconnected nanoparticle clusters	75.8% (635 nm)	142.63	8000 s	This work

## Data Availability

The raw data supporting the conclusions of this article will be made available by the authors on request.

## Supplementary Materials

The following supporting information can be downloaded at: https://www.mdpi.com/article/10.3390/polym18202542/s1, Figure S1: Cross-section images of ESEM images of (a) WO, (b) P-WO, (c) Mn-WO, and (d) P-Mn-WO films; Figure S2: (a,b) After stability FESEM images of P-Mn-WO electrode.
