# Supplementary material for: Engineering the Structure–Performance Relationship of WO3 Thin Films Through PVP–Mn Synergy for Electrochromic Applications
Source: Polymers (Basel). 2026 Oct 16;18(20):2542. doi: 10.3390/polym18202542 (PMC13708851; doi:10.3390/polym18202542)
Supplement: Supplementary file 1 [file polymers-18-02542-s001.zip › polymers-4571150-supplementary.pdf]

Supplementary file

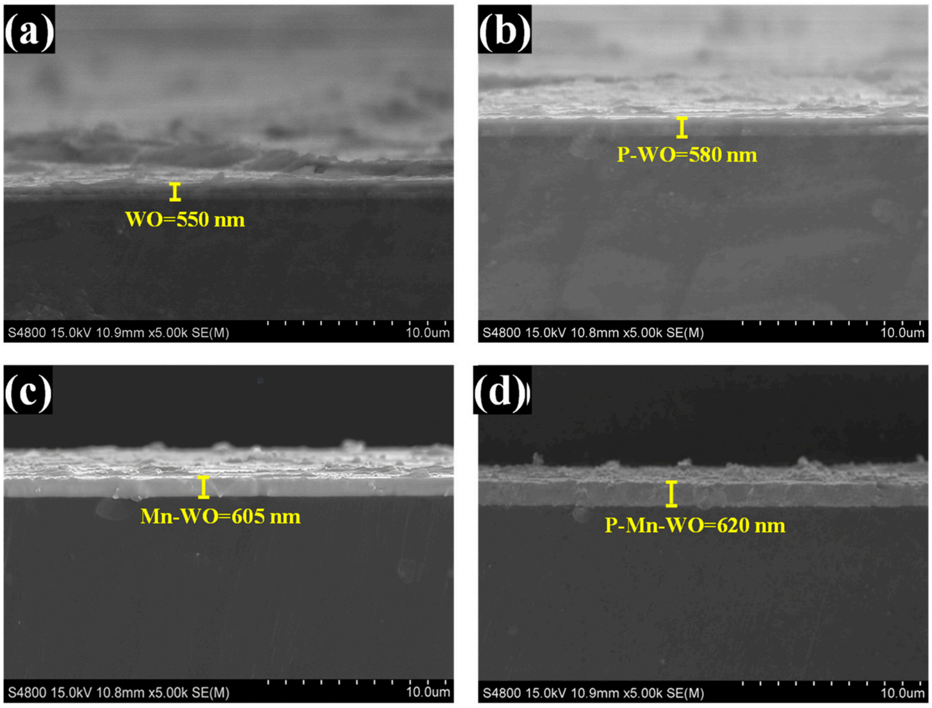

Figure S1: Cross-section images of ESEM images of (a) WO, (b) P-WO, (c) Mn-WO, and (d) P-Mn-WO films.

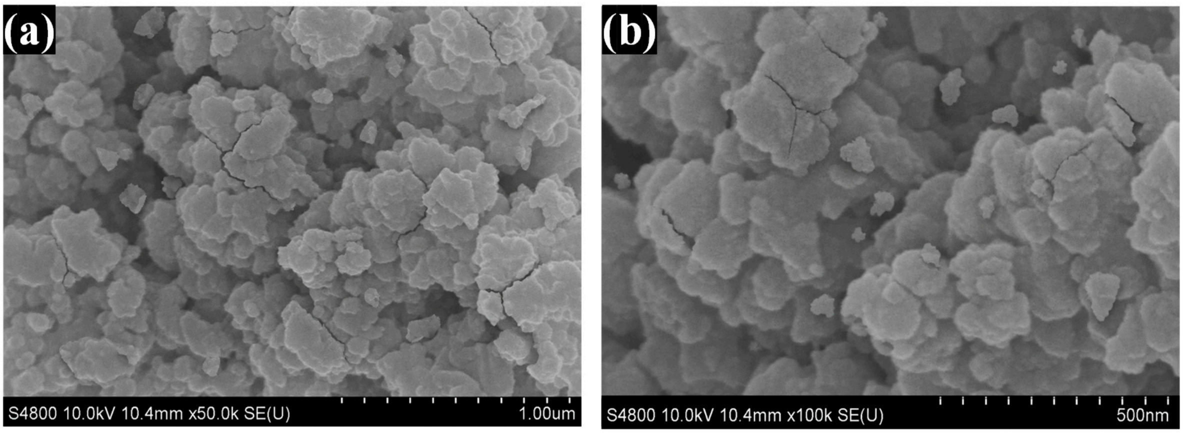

Fig. S2: (a, b) After stability FESEM images of P-Mn-WO electrode.
